# 6-methylflavone exerts selective antimycobacterial activity with membrane energetics disruption and demonstrates therapeutic potential against *Mycobacterium abscessus* infection

**DOI:** 10.1128/spectrum.03588-25

**Published:** 2026-04-20

**Authors:** Jaehun Oh, Jeong-Won Shin, Hyejun Seo, Hyung-Jun Kim, Nam-Jung Kim, Bum-Joon Kim

**Affiliations:** 1 Department of Microbiology and Immunology, College of Medicine, Seoul National University 37990, Seoul, Republic of Korea; 2 Department of Biomedical Sciences, College of Medicine, Seoul National University 37990, Seoul, Republic of Korea; 3 College of Pharmacy, Kyung Hee University 26723, Seoul, Republic of Korea; 4 Cancer Research Institute, College of Medicine, Seoul National University 65492, Seoul, Republic of Korea; 5 Division of Pulmonary and Critical Care Medicine, Department of Internal Medicine, Seoul National University Bundang Hospital 541267, Seongnam, South Korea; 6 Department of Internal Medicine, Seoul National University College of Medicine 198707 , Seoul, South Korea; 7 Department of Life and Nanopharmaceutical Sciences, Graduate School, Kyung Hee University 26723, Seoul, South Korea; 8 Medical Research Center, Institute of Endemic Diseases, Seoul National University 26725, Seoul, Republic of Korea; 9 Wide River Institute of Immunology, Seoul National University, Hongcheon, Republic of Korea; University of Nebraska Medical Center, Omaha, Nebraska, USA

**Keywords:** nontuberculous mycobacteria, *Mycobacterium abscessus*, 6-methylflavone, flavonoids, narrow-spectrum antibiotics, membrane energetics disruptors

## Abstract

Treatment of nontuberculous mycobacteria (NTM) diseases, especially *Mycobacterium abscessus* pulmonary disease, remains a major clinical challenge due to its broad antibiotic resistance and limited therapeutic options. To search for new candidates, we established a synthetic flavonoid library and screened it using a bioluminescent *M. abscessus* reporter strain. Among the compounds tested, 6-methylflavone (6-MF) was identified as a selective inhibitor of mycobacterial growth. 6-MF showed consistent activity against multiple clinical isolates of *M. abscessus* with minimum inhibitory concentration (MIC) values of 8–16 μg/mL and was also effective against intracellular mycobacteria without cytotoxicity. The antimicrobial activity was largely restricted to mycobacteria, indicating its narrow spectrum. Checkerboard assays demonstrated strong synergy with clarithromycin, while the effect with amikacin was limited to weak synergy or indifference. Resistance to 6-MF emerged at a much lower frequency than to amikacin in vitro, underlining its clinical advantage. Notably, 6-MF increased mycobacterial respiratory flux and reduced membrane potential, phenotypes associated with disruption of membrane energetics. In a mouse model of pulmonary infection, 6-MF reduced bacterial burden and lung inflammation to a level comparable to amikacin. These results indicate that 6-MF is a promising lead compound with the potential for further development as a therapeutic option for *M. abscessus* infection.

**IMPORTANCE:**

Despite the increasing global incidence of nontuberculous mycobacterial (NTM) diseases, effective treatment regimens remain limited. In particular, *Mycobacterium abscessus* exhibits notoriously multidrug resistance, making its treatment extremely challenging. In this study, we identified 6-methylflavone (6-MF) as a selective antimycobacterial compound through screening of synthetic flavonoid library. 6-MF specifically inhibited mycobacterial growth and disrupted membrane energetics while showing negligible effects on other bacterial genera. Mycobacteria have unique energy metabolic systems distinct to other bacteria and have been reported to be particularly susceptible to energy metabolism disruptors, such as bedaquiline, clofazimine, and telacebec (Q203). We hope this research will inspire further studies aimed at developing novel antimycobacterial strategies based on energetics disruption.

## INTRODUCTION

*Mycobacterium* constitutes a large genus of bacteria that are ubiquitously distributed in diverse environments, including soil and water. While most mycobacterial species are non-pathogenic, *Mycobacterium tuberculosis* and *Mycobacterium leprae* are recognized as true pathogens, and certain nontuberculous mycobacteria (NTM) have been reported as clinically significant opportunistic pathogens. The treatment success rate for tuberculosis has been improved with the development of effective antituberculous regimens, and the introduction of bedaquiline, pretomanid, and linezolid (BpaL) regimen has significantly enhanced outcomes in the treatment of multidrug-resistant tuberculosis (MDR-TB) (1, 2). However, the treatment success rate for NTM diseases has remained stagnant, primarily due to the lack of effective treatment regimens and increasing antibiotic resistance (3). Among these pathogens, *M. abscessus* exhibits exceptionally high resistance to many antibiotics, posing substantial therapeutic challenges (4). In fact, cure rates for *M. abscessus* pulmonary infections typically range between 41% and 46% (5).

Natural compounds have emerged as attractive candidates for novel antibiotics due to their low toxicity, structural diversity, and potent activity against drug-resistant bacteria. The exploration of natural compounds as potential antibiotic agents offers promise in mitigating the increasing threat of NTM infections (6, 7).

Flavonoids are a class of natural polyphenolic compounds ubiquitously found across diverse plant species. These compounds have been reported to possess a wide range of biological activities, including anti-inflammatory, anticancer, and antimicrobial properties (8–10). Consequently, increasing attention has been directed toward exploring their potential therapeutic applications in the treatment of human diseases. The core structure of flavonoids consists of two phenyl rings interconnected by a heterocyclic ring, and their biological activities are closely associated with specific structural configurations and the nature of the chemical substituents present (11).

In this study, a flavonoid library was established through organic synthesis, and a bioluminescent reporter system was employed to screen the library for antimicrobial activity, with the aim of discovering a novel antibiotic agent for the treatment of NTM diseases. Through this process, we identified 6-methylflavone as a novel antimicrobial agent that specifically targets mycobacteria, and we evaluated its therapeutic potential in a murine model of pulmonary *M. abscessus* infection.

## RESULTS

### Flavonoid library synthesis and screening strategy

**Fig 1:**
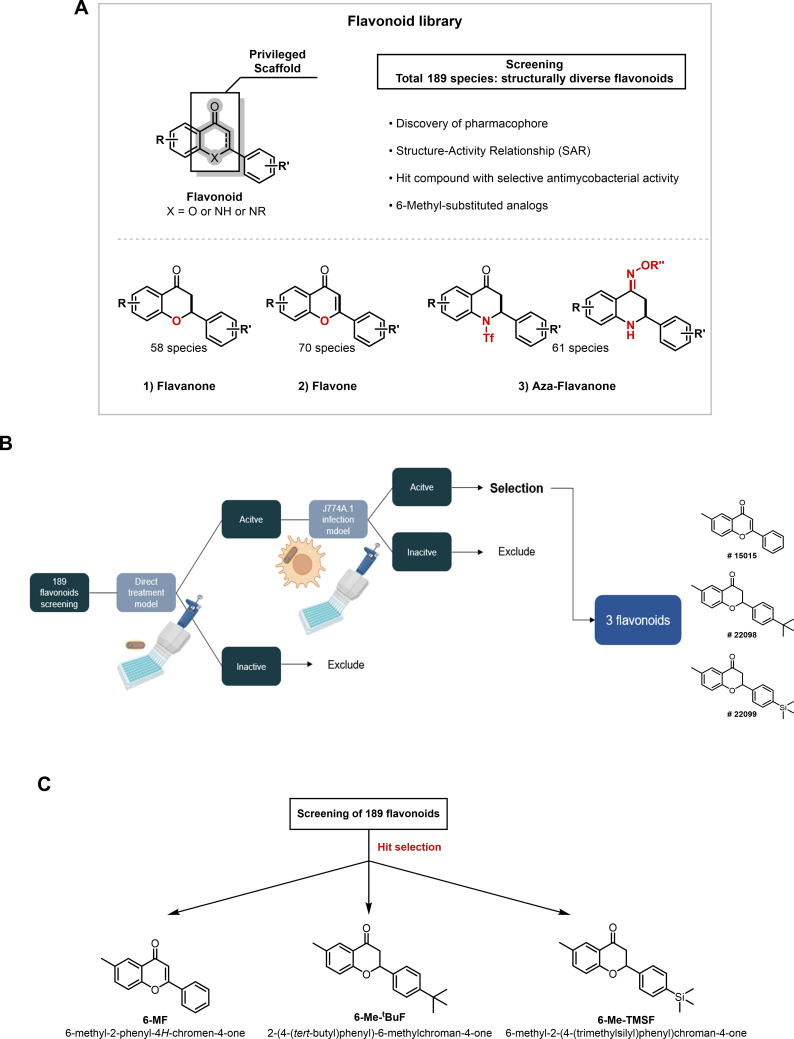
Synthetic and screening strategy of flavonoid library. (**A**) The synthetic strategy of the flavonoid library consisting of 189 flavonoids. (**B**) A total of 189 flavonoids was investigated for their antimycobacterial activity against *M. abscessus* using bioluminescence. The entire library was assessed at a single dose of 20 μM against a bioluminescent clinical isolate of *M. abscessus* using two distinct approaches: direct exposure to mycobacteria and exposure to intracellular mycobacteria in the mouse macrophage cell line J774A.1. Ultimately, 6-MF, 6-Me-tBuF, and 6-Me-TMSF were selected based on their performance in these assays. (**C**) Chemical structure and IUPAC nomenclature of 6-MF, 6-Me-tBuF, and 6-Me-TMSF.

The synthetic routes for flavonoids including flavones, flavanones, and aza-flavanones have been reported in our previous works. Using these synthetic methods, the flavonoid library comprising 189 flavonoid analogs was constructed. Furthermore, structure-activity relationship (SAR) studies based on the screening were performed to identify the pharmacophores and functional groups critical for their antimicrobial activity (Fig. 1A).

Screening system using a bioluminescent *M. abscessus* strain was established for use as a reporter for quantifying bacterial viability, as described in our previous work (12, 13). The recombinant strain was generated by introducing the pMV306_luxG13 plasmid into clinical isolates of *M. abscessus* (Mab_luxG13), and luminescence was measured using a Tecan F200 microplate reader (Fig. S1A). All bioluminescent mycobacterial strains exhibited a linear and positive correlation between OD_600_ and luminescence (Fig. S1B). Furthermore, treatment with an antibiotic agent led to a dose-dependent reduction in luminescence (Fig. S1C). These results demonstrate the feasibility of utilizing bioluminescence as a reliable system for antibiotic screening.

Initially, a total of 189 flavonoids were screened for their antimycobacterial activity against Mab_luxG13 #1 via direct exposure to the bacterial culture, and flavonoids exhibiting less than 90% growth inhibition were excluded, with a 90% growth inhibition hit cutoff. The selected compounds were further evaluated using the J774A.1 infection model, and those that failed to achieve the 90% inhibition threshold were similarly excluded. Ultimately, one flavone (compound #15,015) and two flavanones (compounds #22,098 and #22,099) were identified as hit compounds through this comprehensive procedure (Fig. 1B). The IUPAC nomenclature of compounds #15,015, #22,098, and #22,099 is 6-methyl-2-phenyl-4*H*-chromen-4-one, 2-(4-(*tert*-butyl)phenyl)-6-methylchroman-4-one, and 6-methyl-2-(4-(trimethylsilyl)phenyl)chroman-4-one, respectively. For ease of reference, these compounds are abbreviated as 6-methylflavone (6-MF), 6-methyl-4′-(tert-butyl)flavanone (6-Me-tBuF), and 6-methyl-4′-(trimethylsilyl)flavanone (6-Me-TMSF) in the present work (Fig. 1C).

### 6-MF, 6-Me-tBuF, and 6-Me-TMSF inhibit growth of *Mycobacterium abscessus*

**Fig 2:**
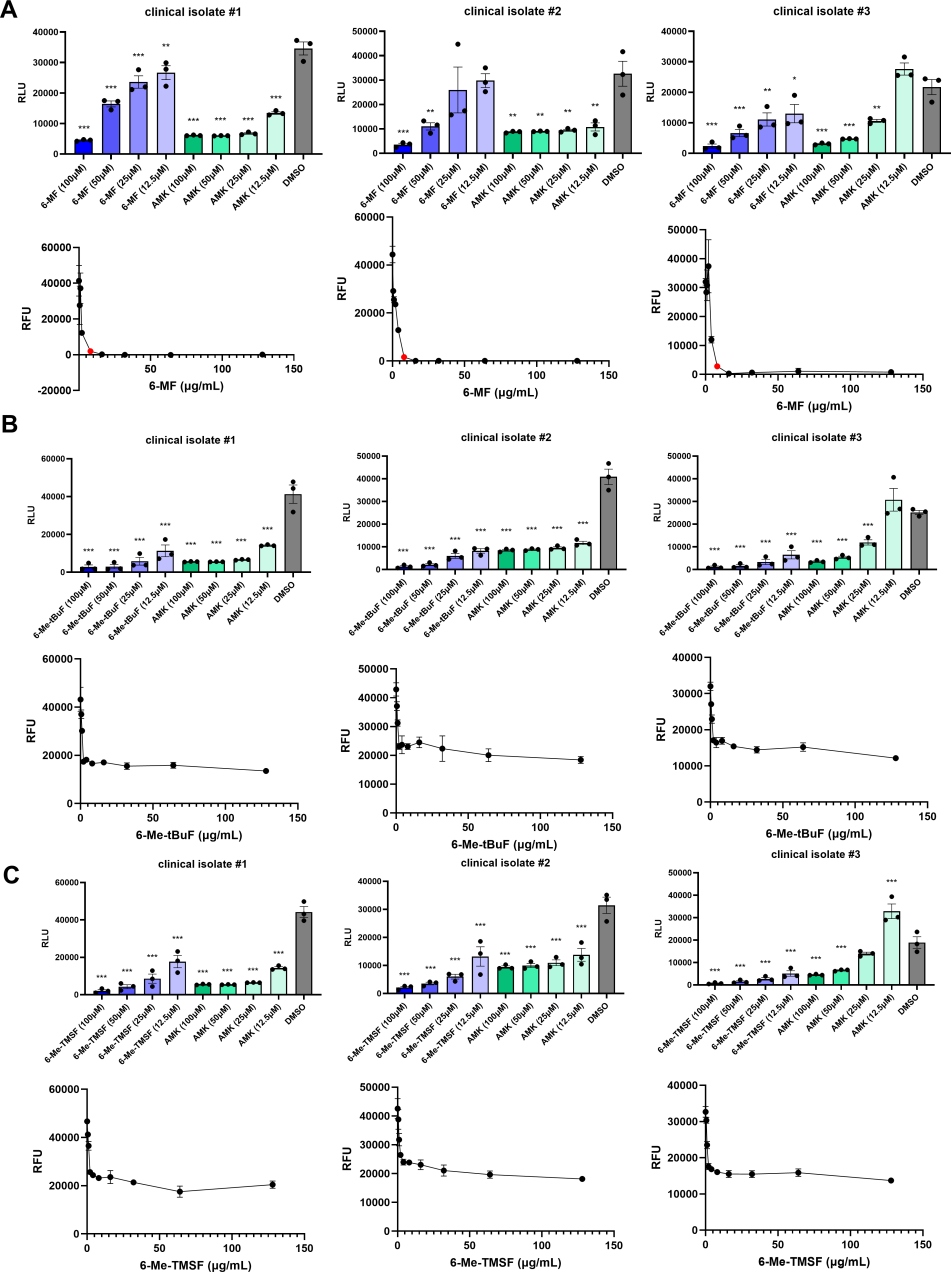
Antimycobacterial activity of 6-MF, 6-Me-tBuF, and 6-Me-TMSF against *M. abscessus*. (**A**, Upper panel) 6-MF was treated to *M. abscessus* growth culture (5 × 10^6^ CFU/mL) in a dose-dependent manner. The cultures were incubated for 24 h, after which luminescence was measured using a Tecan F200 microplate reader (*n* = 3). (**A**, Lower panel) 6-MF was treated to *M. abscessus* growth culture (2.5 × 10^4^ CFU/mL) in a dose-dependent manner as depicted in the figures. The cultures were incubated for 72 h, after which resazurin was added to each well at a final concentration of 15 μg/mL. Fluorescence was measured at ex540/em590 nm following a 2-hour incubation (*n* = 3). MIC was depicted as a red dot in the graph. (**B**, Upper panel) 6-Me-tBuF was treated to *M. abscessus* growth culture (5 × 10^6^ CFU/mL) in a dose-dependent manner. The cultures were incubated for 24 h, after which luminescence was measured using a Tecan F200 microplate reader (*n* = 3). (**B**, Lower panel) 6-Me-tBuF was treated to *M. abscessus* growth culture (2.5 × 10^4^ CFU/mL) in a dose-dependent manner as depicted in the figures. The cultures were incubated for 72 h, after which resazurin was added to each well at a final concentration of 15 μg/mL. Fluorescence was measured at ex540/em590 nm following a 2-hour incubation (*n* = 3). (**C**, Upper panel) 6-Me-TMSF was treated to *M. abscessus* growth culture (5 × 10^6^ CFU/mL) in a dose-dependent manner. The cultures were incubated for 24 h, after which luminescence was measured using a Tecan F200 microplate reader (*n* = 3). (**C**, Lower panel) 6-Me-TMSF was treated to *M. abscessus* growth culture (2.5 × 10^4^ CFU/mL) in a dose-dependent manner as depicted in the figures. The cultures were incubated for 72 h, after which resazurin was added to each well at a final concentration of 15 μg/mL. Fluorescence was measured at ex540/em590 nm following a 2-hour incubation (*n* = 3). All experiments were conducted in triplicate, and error bars represented the SEM. Statistical significance was determined by one-way ANOVA with Tukey’s multiple comparison test. The statistical significance is indicated only for comparison versus DMSO, and the results are denoted as follows: *P < 0.05, **P < 0.01, and ***P < 0.001.

For a more comprehensive assessment of the antimycobacterial activity, the compounds were tested against three distinct clinical isolates—Mab_luxG13, #1, #2, and #3—isolated from different patients. The information about clinical isolates used in the screening is available in the supplemental materials (Table S1). In parallel, MICs were also determined using a resazurin microtiter assay. 6-MF effectively reduced the viability of all three clinical isolates of *M. abscessus* in a dose-dependent manner, although its activity was lower than that of amikacin at the same concentration (Fig. 2A). Consistently, 6-MF inhibited the growth of all clinical isolates of *M. abscessus*, with an MIC value of 8 μg/mL. Both 6-Me-tBuF and 6-Me-TMSF also decreased the viability of all three clinical isolates in a manner similar to 6-MF, and their activities were comparable to, or even greater than, that of amikacin (Fig. 2B and C). However, unlike 6-MF, the growth inhibition by 6-Me-tBuF and 6-Me-TMSF could not reach 90% even at the highest concentration tested, although clear inhibitory effects were still observed. Because maintaining blood concentrations at or above the MIC is essential to achieve therapeutic efficacy in infectious diseases, both 6-Me-tBuF and 6-Me-TMSF, which failed to reach the MIC within their soluble concentration ranges, were excluded from further studies (14, 15).

### 6-MF efficiently inhibits growth of intracellular *M. abscessus* without cytotoxicity

Most pathogenic NTMs are intracellular pathogens that require efficient cellular penetration of antibiotics for effective treatment (16, 17). For this reason, amikacin, which is frequently used for NTM infections, exhibits limited efficacy against intracellular bacteria. In fact, liposome encapsulation of amikacin has been reported in numerous literatures to greatly enhance its intracellular activity (18–20).

**Fig 3:**
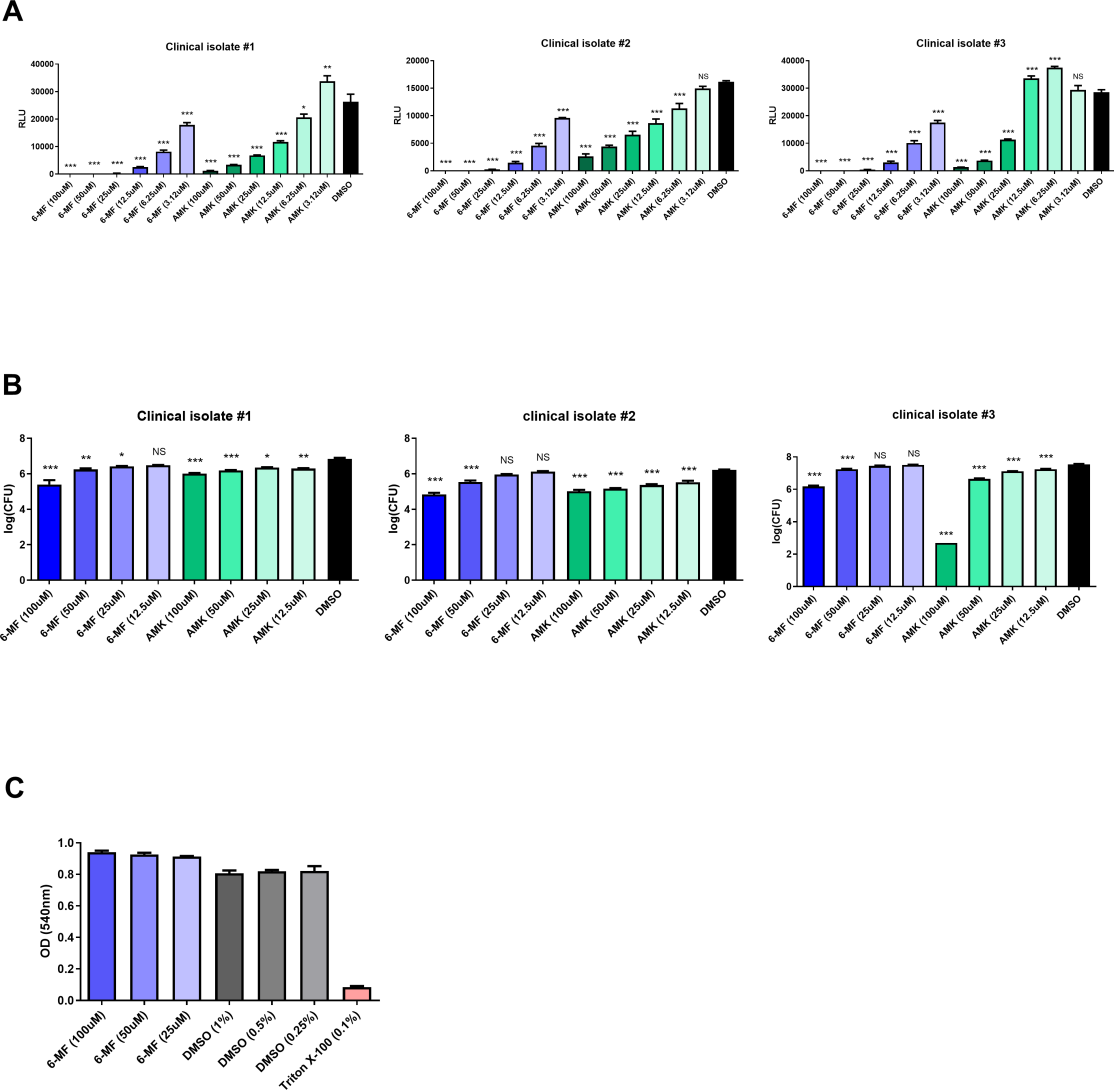
Antimycobacterial activity of 6-MF against intracellular *M. abscessus* and cytotoxicity assessment of 6-MF determined by the neutral red uptake assay. (**A**) J774A.1 cells were infected with bioluminescent *M. abscessus* with MOI of 10 for 2 h. Subsequently, cells were washed with PBS and treated with 50 μg/mL amikacin for 1 h to eliminate extracellular mycobacteria. After washing, freshly prepared 6-MF was applied to the J774A.1 cells. Luminescence was measured 24 h post-infection using a Tecan F200 microplate reader. (**B**) J774A.1 cells were infected with bioluminescent *M. abscessus* with MOI of 10 for 2 h. Subsequently, cells were washed with PBS and treated with 50 μg/mL amikacin for 1 h to eliminate extracellular mycobacteria. After washing, freshly prepared 6-MF was applied to the J774A.1 cells. J774A.1 cells were lysed using 1% Triton X-100 48 h post-infection, and the lysates were plated on 7H10 agar plates for CFU assay. The statistical significance is indicated only for comparison versus DMSO. (**C**) 6-MF was treated to J774A.1 for 48 h at concentrations of 25–100 μM. Subsequently, the J774A.1 cells were stained with neutral red for 2 h, followed by destaining and measurements of OD_540_. All experiments were conducted in triplicate, and error bars represent the SEM. Statistical significance was determined by one-way ANOVA with Tukey’s multiple comparison test, and the results are denoted as follows: *P < 0.05; **P < 0.01; ***P < 0.001; NS, not significant.

Therefore, the antimycobacterial activity of 6-MF was further evaluated in a J774A.1 macrophage infection model using three Mab_luxG13 strains. 6-MF efficiently and dose-dependently reduced the viability of intracellular *M. abscessus*. Moreover, the luminescence reduction induced by 6-MF was greater than that induced by amikacin at the same concentration (Fig. 3A), in contrast to the result observed under direct exposure conditions (Fig. 2A). To corroborate these findings, a colony-forming unit (CFU) assay was performed to quantify viable intracellular bacteria. However, unlike the luminescence-based results, the reduction in CFU was superior in cells treated with amikacin than in those treated with 6-MF (Fig. 3B). This discrepancy between luminescence- and CFU-based results may indicate a bacteriostatic profile for 6-MF, in contrast to the strong bactericidal activity of amikacin. Furthermore, a neutral red uptake (NRU) assay demonstrated that 6-MF did not affect the viability of J774A.1 cells (Fig. 3C), indicating that the observed reductions in luminescence and CFU were not attributable to cytotoxicity.

Collectively, these results demonstrate that 6-MF exhibits potent inhibitory activity against both intracellular and extracellular *M. abscessus*.

### 6-MF exhibits selective antimicrobial activity against *Mycobacterium* species and displays a bacteriostatic profile

To further assess the antimicrobial spectrum of 6-MF, we determined its MICs against various *Mycobacterium* species. Against rapidly growing mycobacteria (RGM), MICs of 6-MF were 8 μg/mL for *M. chelonae* subsp. *bovistauri*, 2 μg/mL for *M. chelonae* subsp. *gwanakae*, 8 μg/mL for *M. fortuitum*, and 8 μg/mL for *M. smegmatis*. In contrast, against slowly growing mycobacteria (SGM), 6-MF exhibited MICs of 32 μg/mL for *M. avium*, 16 μg/mL for *M. bovis* BCG, 32 μg/mL for *M. intracellulare*, 32 μg/mL for *M. intracellulare* subsp. *yongonense*, 32 μg/mL for *M. marinum*, 16 μg/mL for *M. paragordonae*, and 16 μg/mL for *M. paraintracellulare* (Table 1). Overall, 6-MF showed lower MICs against RGM compared to SGM.

**TABLE 1:** MICs of 6-MF against various mycobacteria

Species	Strain	MIC (μg/mL)
*M. chelonae* subsp. *bovistauri*	QIA-37	8
*M. chelonae* subsp. *gwanakae*	MOTT36W	2
*M. fortuitum*	ATCC 6841	8
*M. smegmatis*	mc^2^155	8
*M. avium*	ATCC 25291	32
*M. bovis* BCG	BCG Tokyo	16
*M. intracellulare* subsp. *intracellulare*	ATCC 13950	32
*M. intracellulare* subsp. *yongonense*	05-1390^T^	32
*M. marinum*	ATCC 927	32
*M. paragordonae*	49061^T^	16
*M. paraintracellulare*	MOTT64	16

The antimicrobial activity of 6-MF was also evaluated against bacterial species and yeast other than the genus *Mycobacterium* (Fig. S2). 6-MF rarely inhibited the growth of these non-mycobacterial species, even at its maximum soluble concentration, suggesting that 6-MF exhibits *Mycobacterium*-specific inhibitory activity. Interestingly, although 6-MF did not achieve complete inhibition of *Candida albicans*, it partially reduced its growth in a dose-dependent manner, unlike other bacterial species.

To investigate whether the antimycobacterial activity of 6-MF is associated with the basic flavone core structure, 6-MF and basic flavone were compared for their inhibitory activity on the growth of *M. abscessus*. Although basic flavone also exhibited inhibitory activity, its effect was obviously weaker than that of 6-MF, suggesting that the methyl group at the C6 position plays an important role in the antimycobacterial activity (Fig. S3).

The MIC and minimal bactericidal concentration (MBC) of 6-MF against clinical isolates of *M. abscessus* were also determined to establish whether it exhibits bacteriostatic or bactericidal activity. The values for the MIC and MBC against eight clinical isolates are listed in Table 2. The MIC range of 6-MF against clinical isolates was found to be 8–16 μg/mL. However, the MBC of 6-MF against clinical isolates was notably higher than the MIC, with a ratio exceeding four, except for one strain. This discrepancy may arise from strain-dependent factors, such as metabolic state, efflux capacity, membrane permeability, or efficiency in compensatory responses.

**TABLE 2:** MIC, MBC, and MBC/MIC ratio of 6-MF against clinical isolates of *M. abscessus*

	MIC (μg/mL)	MBC (μg/mL)	MBC/MIC ratio	Type
*M. abscessus* Smooth #1	16	256	16	Bacteriostatic
*M. abscessus* Smooth #2	8	16	2	Bactericidal
*M. abscessus* Rough #1	16	512	32	Bacteriostatic
*M. abscessus* Rough #2	16	>512	>32	Bacteriostatic
*M. massiliense* Smooth #1	8	256	32	Bacteriostatic
*M. massiliense* Smooth #2	16	256	16	Bacteriostatic
*M. massiliense* Rough #1	8	256	32	Bacteriostatic
*M. massiliense* Rough #2	8	> 512	>64	Bacteriostatic

These results suggest that 6-MF exerts its effect through a bacteriostatic mechanism rather than a bactericidal one (21, 22). The bacteriostatic profile of 6-MF may explain the differing patterns observed in luminescence and CFU reductions compared with amikacin.

### 6-MF exhibits a synergistic effect with clarithromycin on antimicrobial activity against *Mycobacterium abscessus*

Given that the standard treatment for *M. abscessus* infection recommends a combination of regimens, the presence of synergistic effect with other antibiotics becomes an attractive property for potential candidates in *M. abscessus* infection treatment (23).

Therefore, the potential synergistic effects of 6-MF with amikacin and clarithromycin, two commonly used antibiotics for the treatment of *M. abscessus* infection, were evaluated through checkerboard assay. This assay was performed in both a direct exposure model and a J774A.1 infection model with Mab_luxG13 derived from clinical isolate 1. Synergy was evaluated based on the fractional inhibitory concentration index (FICI) in the direct exposure model and using ZIP, Bliss, Loewe, and HSA models in the infection model via SynergyFinder web application.

**Fig 4:**
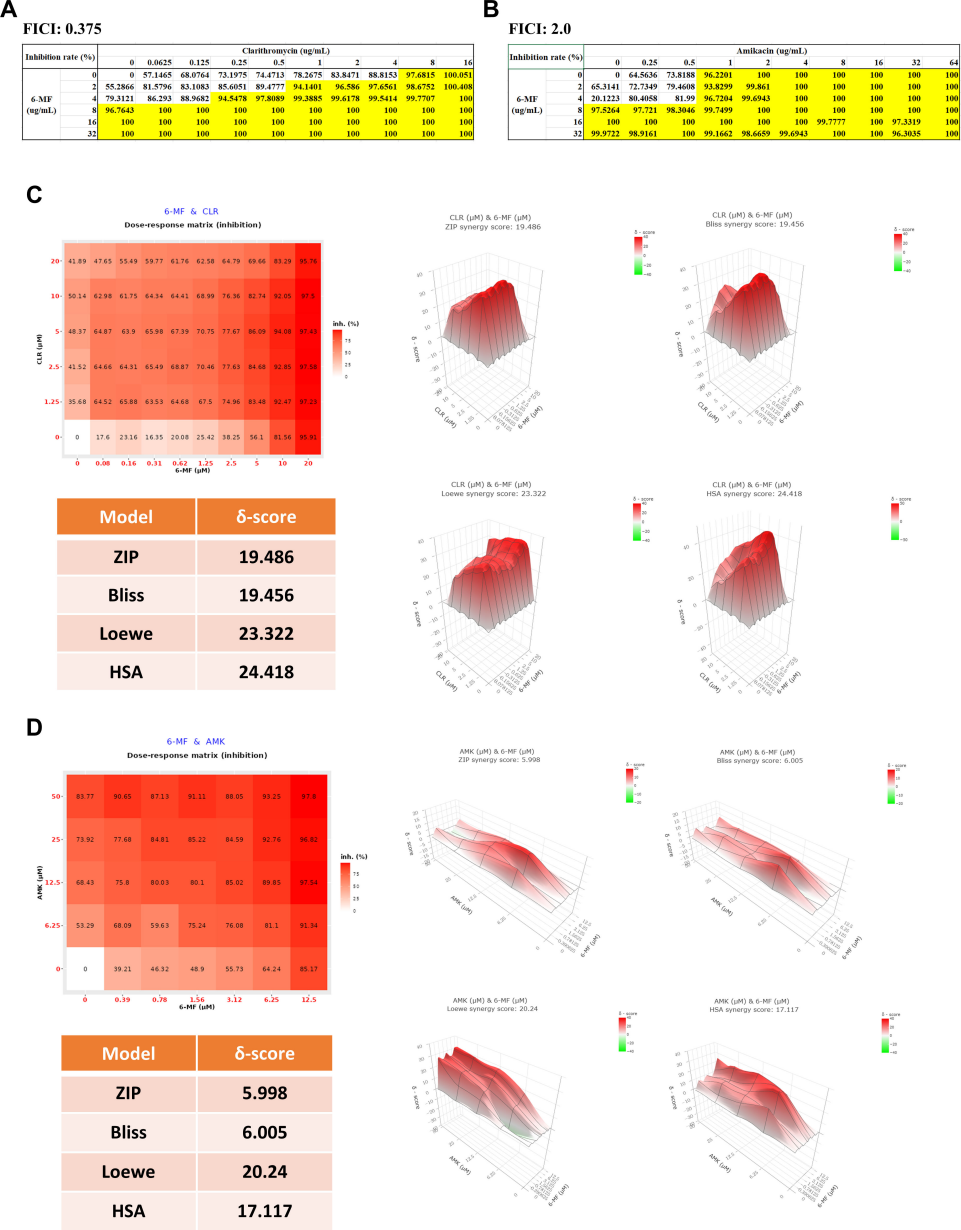
Synergistic effect of 6-MF against clinical isolate of *M. abscessus*. Clarithromycin (**A**) or amikacin (**B**) was co-administered with 6-MF in a dose-dependent manner to *M. abscessus* growth culture. OD_600_ was measured at 72 h using a Tecan F200 microplate reader. Drug combinations that exhibited ≥90% growth inhibition were indicated as yellow boxes in the table. Clarithromycin (**C**) or amikacin (**D**) was co-administered with 6-MF to J774A.1 cells infected with bioluminescent *M. abscessus*. Luminescence was measured 24 h post-infection, and the measured luminescence values were calculated to inhibition rate and analyzed for the presence of synergism using ZIP, Bliss, Loewe, and HSA mathematical synergy model. The inhibition rate by dose was represented with a color spectrum from white (low) to red (high), and the synergy score by dose was represented with a color spectrum from green (low) to red (high).

The result demonstrated strong synergism between 6-MF and clarithromycin. The FICI value of 0.375 met the standard criterion for synergy (Fig. 4A). In addition, synergy scores obtained from all four models were close to 20, indicating robust synergistic effect against intracellular *M. abscessus* (Fig. 4C). Importantly, clinical isolate 1 harbors the *erm* (41) gene, which mediates inducible resistance against macrolides (Fig. S1). Despite this inherent clarithromycin resistance, the addition of clarithromycin markedly enhanced the antimicrobial activity of 6-MF.

In contrast, the synergistic interaction between 6-MF and amikacin was less definitive. The FICI value was 2, indicating indifference (Fig. 4B). However, in the J774A.1 infection model, synergy scores were ZIP: 5.998, Bliss: 6.005, Loewe: 20.24, and HSA: 17.117, suggesting some degree of synergism (Fig. 4D). While the Loewe and HSA models suggested strong synergistic effects, ZIP and Bliss indicated only weak or modest synergy. Considering that the Loewe model is more suitable when the two drugs share a similar mechanism of action, and the HSA model tends to be overly simplistic and optimistic, the ZIP and Bliss models are regarded as more reliable in this context (24).

Altogether, these results indicate that 6-MF exhibits a strong synergistic inhibitory activity with clarithromycin and only weak synergy or indifference with amikacin against *M. abscessus*, emphasizing its promising therapeutic potential for the treatment of *M. abscessus* infections.

### *M. abscessus* develops resistance to 6-MF at a low frequency under laboratory conditions

Development of resistance is one of the major obstacles in a novel antibiotic development, as it diminishes their therapeutic utility and deteriorates the revenue structure in market (25, 26). Therefore, low frequency of resistance development is an important property in antibiotics.

**Fig 5:**
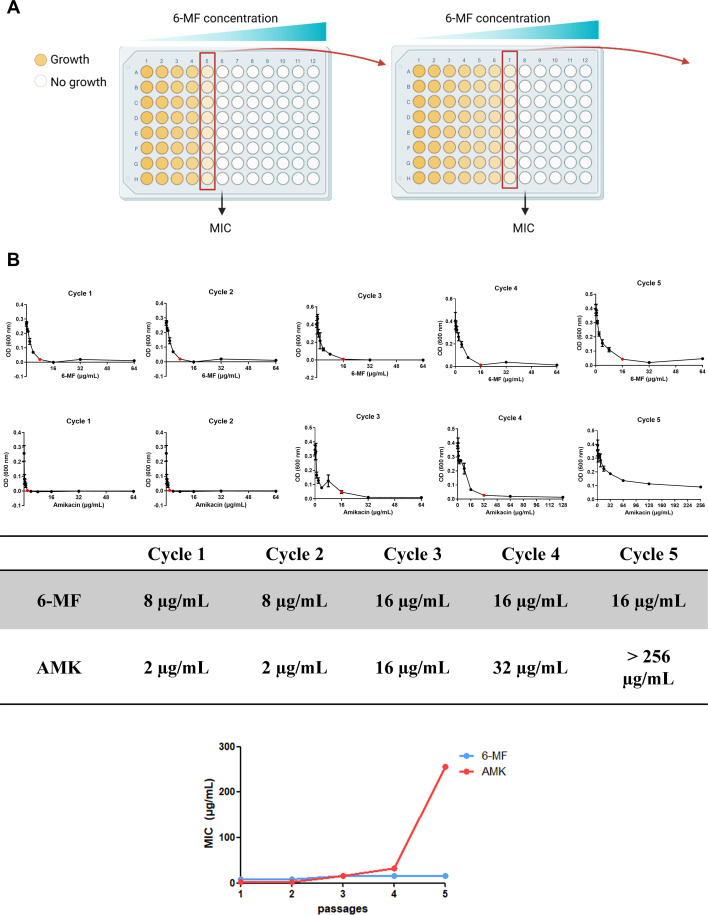
Adaptive laboratory evolution assay for resistance induction in *M. abscessus*. (**A**) Schematic representation of adaptive laboratory evolution assay. A broth microdilution assay was performed according to CLSI guidelines, and the plates were incubated at 37°C for 7 days. Cultures from 1/2 × MIC wells were harvested and transferred to the next cycle. (**B**) The cycles were repeated up to cycle 5, and the MIC values of each cycle were recorded. MICs are shown as red dots in the graphs. All experiments were conducted in triplicate, and error bars represent the SEM.

To assess the propensity for resistance development in *M. abscessus*, an adaptive laboratory evolution assay using a gradient approach was performed with 6-MF (Fig. 5A). The MIC of amikacin increased exponentially with successive cycles, reaching 128-fold higher than the original MIC by cycle 5. In contrast, the MIC of 6-MF increased only 2-fold over the same number of cycles, indicating a markedly lower tendency for resistance development to 6-MF compared with amikacin (Fig. 5B).

### 6-MF disrupts membrane energetics via membrane potential dissipation in *M. abscessus*

**Fig 6:**
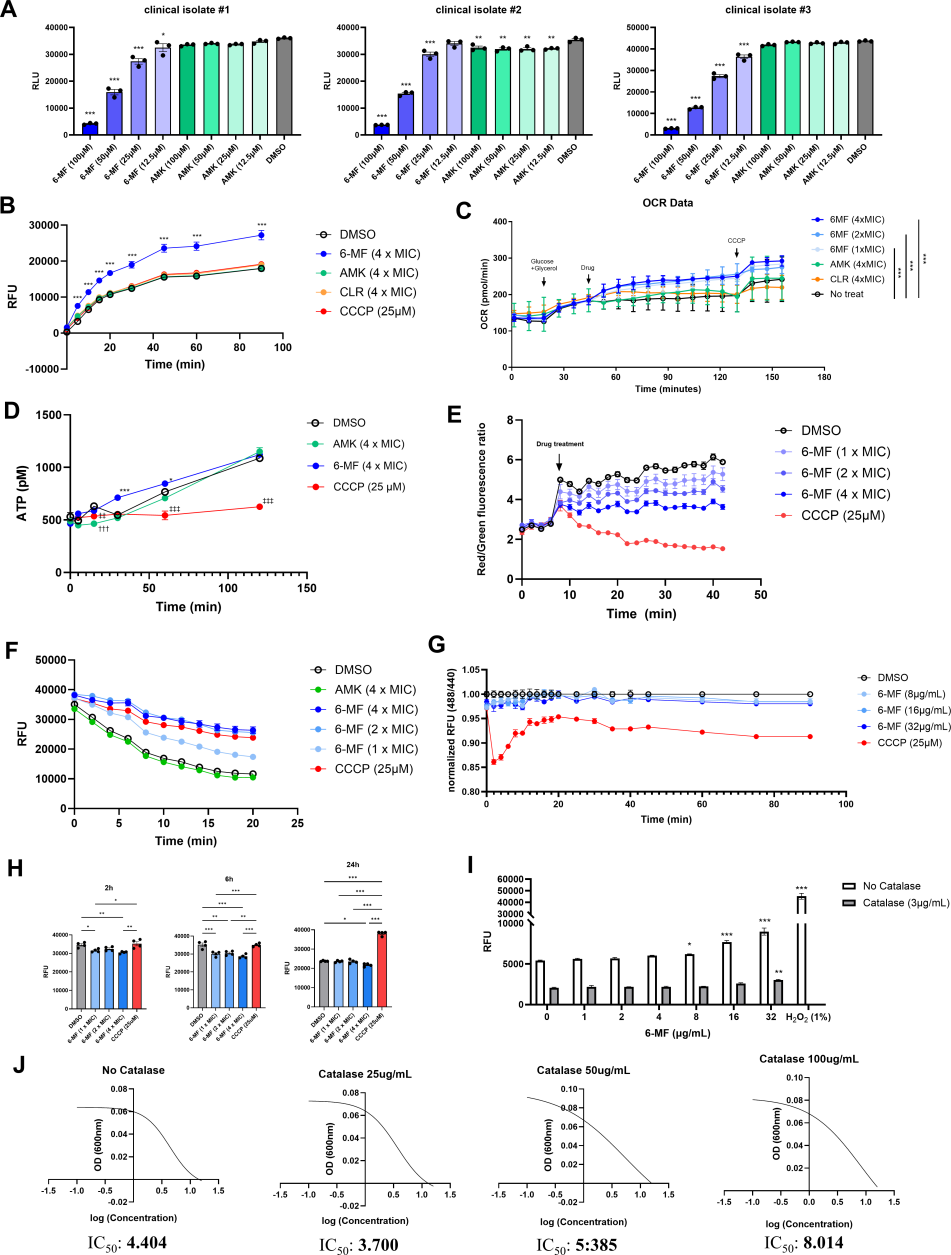
Alteration of energetics upon 6-MF-induced membrane potential dissipation in *M. abscessus* (**A**) Luminescence of Mab_luxG13 cultures was measured immediately after treatment with 6-MF or amikacin (*n* = 8). The statistical significance is indicated only for comparison versus DMSO. (**B**) *M. abscessus* ATCC 19977 cultures were treated with 4 × MIC of each drug (8 μg/mL for amikacin, 2 μg/mL for clarithromycin, and 32 μg/mL for 6-MF) and CCCP (25 μM), followed by the addition of resazurin at a final concentration of 0.15 mg/mL The statistical significance is indicated only for comparison versus DMSO. Fluorescence was monitored at ex540/em590 nm using a Tecan F200 microplate reader (*n* = 3). (**C**) *M. abscessus* ATCC 19977 cells were washed with PBS, resuspended in unbuffered 7H9 medium, and seeded (10^6^ CFU/well) onto poly-D-lysine-coated XF24 culture plates for analysis using a Seahorse XF24e analyzer. The first three measurements were obtained without a carbon source, after which a glucose-glycerol mixture (final concentration of 0.2%) was automatically injected. Following three additional measurements, each drug was injected (*n* = 3). CCCP (2 μM) was injected into each well prior to the final three measurements. (**D**) *M. abscessus* ATCC 19977 cultures were treated with 4 × MIC of each drug, and samples were collected at different time points. Intracellular ATP levels were determined using the Bactiter-Glo microbial cell viability assay kit (Promega) (*n* = 2). The statistical significance is indicated only for comparison versus DMSO with different symbol denoting each treatment group (6-MF, *; AMK, †; CCCP, ‡). (**E**) *M. abscessus* ATCC 19977 was pre-incubated with 30 μM diOC_2_(3) for 40 min and loaded into black 96-well plates. Fluorescence was then measured at ex485/em520 (green) or ex485/em620 nm (red) four times prior to compound addition, followed by consecutive fluorescence measurement after compound addition (*n* = 6). (**F**) *M. abscessus* ATCC 19977 was pre-incubated with 2 μg/mL EtBr for 1 h, washed with PBS, and loaded into black 96-well plate. Fluorescence was then immediately measured at ex530/em590 nm following compound addition (*n* = 8). (**G**) *M. abscessus* ATCC 19977 was pre-incubated with 20 μM BCECF-AM for 30 min and loaded into a black 96-well plate. Compounds were added, and fluorescence was immediately measured at ex440/em535 nm or ex488/em515 nm. (**H**) *M. abscessus* ATCC 19977 was treated with the indicated compounds for 2, 6, and 24 h, followed by staining with 20 μM PI for 15 min in the dark. After washing with PBS, fluorescence was measured at ex535/em617 nm. (**I**) *M. abscessus* ATCC 19977 cultures were incubated in unbuffered 7H9 containing DCFDA at 37°C for 30 min in the dark. Bacterial suspensions were washed three times with PBS and treated with 6-MF in the presence or absence of catalase. Fluorescence was measured 30 min after 6-MF treatment at ex485/em535 nm (*n* = 3). (**J**) MIC_50_ was determined using broth microdilution assay in CAMHB with different concentrations of bovine catalase (*n* = 3). Error bars represent the SEM. Statistical significance was determined by one-way ANOVA or two-way ANOVA with Tukey’s multiple comparison test, and the results are denoted as follows: *, † or ‡ (*P* < 0.05), **, ††, or ‡‡ (*P* < 0.01), and ***, ††† or ‡‡‡ (*P* < 0.001).

An immediate decrease in luminescence was observed in all Mab_luxG13 strains upon 6-MF treatment at the 0 h time point, in contrast to amikacin (Fig. 6A). Given that 6-MF is not a robust bactericidal or disinfectant agent, this rapid reduction likely reflects effects on components required for bacterial luciferase activity. Unlike firefly luciferase, bacterial luciferase depends on FMNH_2_ as substrate, not ATP. We therefore hypothesized that 6-MF affects the production or consumption of reduced redox cofactors.

To test this hypothesis, we measured the reductive potential of *M. abscessus* following 6-MF treatment using resazurin as a redox indicator. Unexpectedly, 6-MF treatment significantly increased the reduction of resazurin to resorufin, suggesting an elevated electron transfer flux (Fig. 6B). To confirm that the elevated electron transfer flux leads to increased respiratory flux, we measured changes in the oxygen consumption rate (OCR) of *M. abscessus* upon 6-MF treatment using a Seahorse XF analyzer. Interestingly, unlike other antibiotics, 6-MF induced a concentration-dependent increase in OCR (Fig. 6C).

Notably, despite the elevated OCR, the increase of ATP was marginal and limited to the initial time points (Fig. 6D). These results suggest that the increased respiration might be a compensatory response to disrupted energy metabolism induced by 6-MF.

To understand why respiratory flux increased without proportional ATP production, we evaluated membrane potential using 3,3′-Diethyloxacarbocyanine (DiOC_2_(3)) staining. 6-MF dose dependently reduced bacterial membrane potential (Fig. 6E), but this reduction was limited and did not progress further, in contrast to the continuous collapse caused by the classical uncoupler carbonyl cyanide *m*-chlorophenyl hydrazone (CCCP). This limited dissipation, combined with preserved ATP levels, suggests a mechanism distinct from complete uncoupling.

Most mycobacterial efflux pumps depend on proton motive force (PMF) for their activity, and ethidium bromide (EtBr) has been identified as a broad substrate of these efflux pumps. Accordingly, EtBr has been widely used to evaluate efflux pump activity and PMF integrity in mycobacteria (27–29). Based on this principle, an EtBr efflux assay was performed following treatment with 6-MF. Consistent with the membrane potential results, 6-MF decreased EtBr efflux in a dose-dependent manner, suggesting a reduction in PMF (Fig. 6F).

A classical protonophore uncoupler, CCCP, collapses PMF by transporting protons into the cell, thereby decreasing both the membrane potential (Δψ) and the proton gradient (ΔpH). Therefore, the intracellular pH of *M. abscessus* was compared following treatment with 6-MF or CCCP using the pH-sensitive dye BCECF-AM. Unlike CCCP, which significantly decreased intracellular pH, 6-MF minimally affected intracellular pH (Fig. 6G). On the other hand, the propidium iodide (PI) uptake assay revealed that 6-MF did not compromise membrane integrity, indicating that the decreased Δψ is not attributable to membrane damage. Rather, 6-MF reduced membrane permeability (Fig. 6H). This result demonstrates 6-MF selectively dissipates membrane potential without influencing ΔpH.

Accelerated electron transport chain can increase electron leakage, commonly leading to the generation of reactive oxygen species (ROS) (30). To determine whether the increased respiratory flux promotes ROS generation, intracellular ROS levels were measured using 2’,7′-Dichlorofluorescein diacetate (DCFDA). 6-MF increased ROS production in a concentration-dependent manner (Fig. 6I), which was abrogated by catalase treatment. However, when we assessed whether ROS contributes to the antimicrobial effect, catalase supplementation only slightly increased MIC₅₀ and did not affect MIC₉₀ (Fig. 6J). These results indicate that ROS is not the primary antimicrobial mechanism, but may have a minor contribution to its antibacterial effect.

Collectively, 6-MF disrupts membrane energetics by dissipating membrane potential, thereby increasing respiratory flux as a compensatory response to reduced energy efficiency. However, 6-MF did not completely collapse PMF or ATP homeostasis, which could be partially restored through compensatory responses.

### 6-MF alleviates pulmonary *M. abscessus* infection in a mouse model

**Fig 7:**
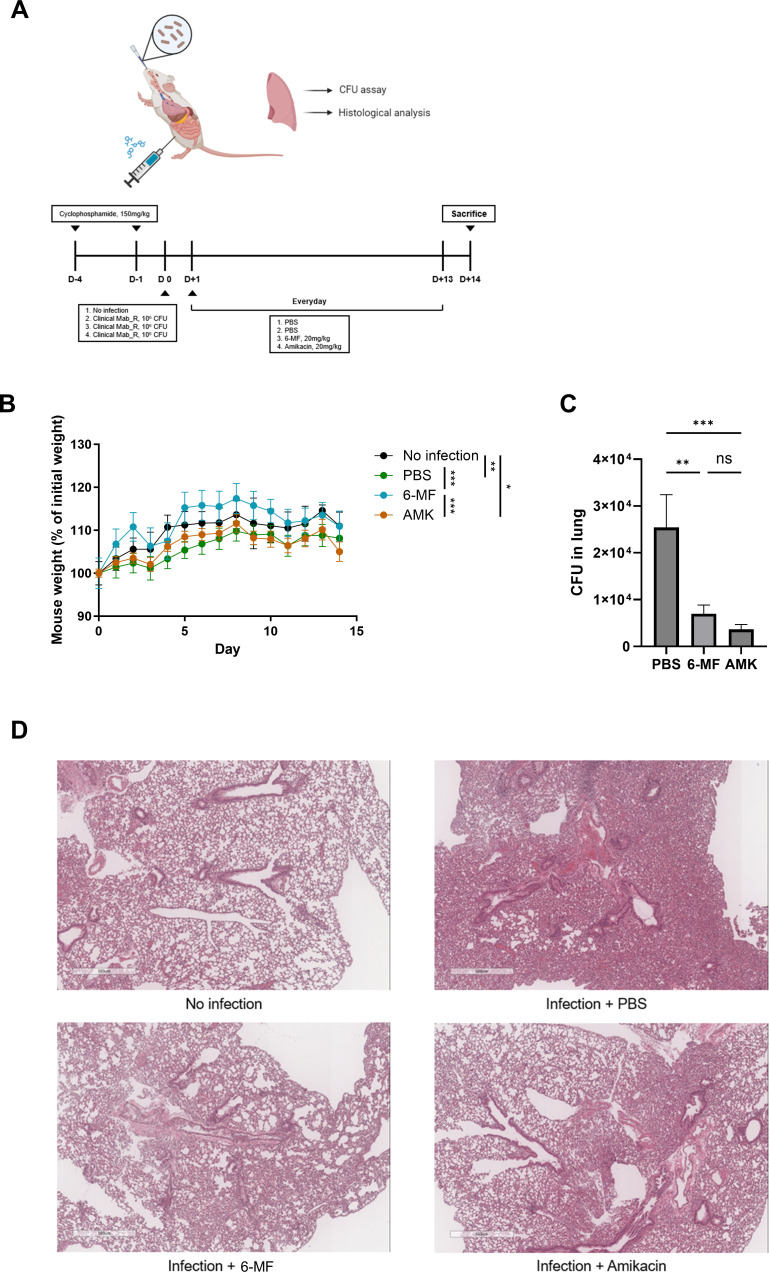
*In vivo* efficacy of 6-MF in the mouse model of pulmonary *M. abscessus* infection. (**A**) Schematic diagram of the experimental schedule. Cyclophosphamide was intraperitoneally administered to mice at a dosage of 150 mg/kg in PBS at day 4 and day 1 prior to the infection. Mice were then inoculated with *M. abscessus* (1 × 10^6^ CFU) via intranasal route. Treatment of 6-MF or amikacin (20 mg/kg) was administered intraperitoneally starting 1 day post-infection and continued daily for 2 weeks. (**B**) Body weight of mice was monitored for 2 weeks post-infection. (**C**) Lungs were homogenated in PBS, and CFU assay was conducted from lung homogenates to assess bacterial burden in lungs. (**D**) The postcaval lobe of the lung was fixed and stained with hematoxylin and eosin, and subsequently observed under a light microscope. Error bars represent the SEM (*n* = 6). Statistical significance was determined by one-way ANOVA or two-way ANOVA with Tukey’s multiple comparison test, and the results are denoted as follows: *P < 0.05; **P < 0.01; ***P < 0.001; ns, not significant.

An *in vivo M. abscessus* mouse model of infection using cyclophosphamide was used to assess the *in vivo* efficacy of 6-MF as an antibiotic agent for the treatment of *M. abscessus* infection (31, 32). BALB/c mice were administered a 150 mg/kg dosage of cyclophosphamide on days 1 and 4 prior to infection to induce a neutropenic state. Subsequently, 10^6^ CFU of *M. abscessus* was challenged via the intranasal route. 6-MF and amikacin were delivered via intraperitoneal route at a daily dosage of 20 mg/kg starting one day after infection and continued for 2 weeks until the mice were sacrificed (Fig. 7A).

The body weight of the 6-MF-treated group exhibited an increase surpassing that of the PBS-treated control group and was comparable to the uninfected control group (Fig. 7B). Additionally, the 6-MF-treated group exhibited a statistically significant reduction in bacterial burden in the lung compared to the PBS-treated control group (Fig. 7C). In accordance with the lung CFU results, histological examination under H&E staining revealed a substantial reduction in lung inflammation in 6-MF-treated groups (Fig. 7D).

## DISCUSSION

The management of NTM diseases, especially infection caused by *M. abscessus,* poses extreme challenges, primarily due to its notorious antibiotic resistance. Consequently, there is an urgent need for the development of novel antibiotics for the treatment of *M. abscessus* infection. In this context, there has been a growing interest in discovering such antibiotics from natural products, including flavonoids, terpenoids, and peptides.

In this study, we identified 6-MF from a synthetic flavonoid library as a selective antimycobacterial compound and demonstrated that it acts as a membrane energetics disruptor, characterized by reduced membrane potential and increased respiratory flux in mycobacteria. 6-MF showed consistent activity against multiple clinical isolates of *M. abscessus*, with MIC values of 8–16 μg/mL, and effectively reduced intracellular bacterial burden in J774A.1 macrophages without cytotoxicity. On the other hand, 6-MF rarely affected the growth of other bacterial genera, indicating its narrow-spectrum antibiotic specificity to mycobacteria.

As the treatment of *M. abscessus* typically involves a combination of multiple antibiotics, the synergistic potential of 6-MF is a valuable property. In our study, 6-MF exhibited strong synergism with clarithromycin in both direct exposure and J774A.1 infection models, as demonstrated with an FICI of 0.375 and high synergy scores (~20) calculated using ZIP, Bliss, Loewe, and HSA models. Importantly, the combination outperformed either monotherapy of 6-MF and clarithromycin even in an *M. abscessus* strain which harbors an intact *erm*(41) gene. This finding suggests the potential use of 6-MF as an adjuvant for macrolide-based therapy in *M. abscessus* infection.

We demonstrated that 6-MF reduces membrane potential and PMF in *M. abscessus*. Consistent with these effects, increased resazurin reduction and elevated oxygen consumption rates indicate that electron transfer and respiratory flux are enhanced as a compensatory response to reduced energy efficiency caused by partial dissipation of membrane energetics. Notably, unlike CCCP, 6-MF did not induce a complete collapse of membrane potential and had minimal effect on intracellular pH. Additionally, intracellular ATP levels were largely maintained. These findings suggest that 6-MF does not fully abrogate energy production but instead perturbs membrane energetics in a manner distinct from classical protonophore uncouplers by selectively dissipating membrane potential.

On the other hand, the resazurin reduction observed under CCCP treatment was comparable to that of the untreated control, despite increased respiratory flux. This likely reflects the persistence of basal redox activity, as reducing equivalents are rapidly consumed by the respiratory chain and fail to accumulate to support enhanced resazurin reduction, highlighting a mechanistic distinction from the compensatory respiratory response induced by 6-MF.

The resistance induction experiment further distinguishes 6-MF from conventional antibiotics. Over five serial adaptive laboratory evolution cycles, the MIC of 6-MF increased only 2-fold, while the MIC of amikacin increased 128-fold. This result indicates that resistance against 6-MF develops at a lower frequency than amikacin.

Preclinical *in vivo* efficacy was also confirmed in a pulmonary *M. abscessus* infection model under cyclophosphamide-induced neutropenic conditions. Daily intraperitoneal administration of 6-MF significantly reduced bacterial burden and lung inflammation and improved body weight recovery in mice to a comparable level of amikacin. These findings demonstrate that 6-MF is active *in vivo* and support its potential use for NTM diseases, although comprehensive clinical research must be preceded.

6-MF has been recognized primarily as a GABA_A_/benzodiazepine receptor antagonist and TAS2R39 blocker rather than an antimicrobial agent (33, 34). However, a limited study has reported its antimicrobial activity against *E. coli* and *Cutibacterium acnes* through a disk diffusion assay, which conflicts with our observation that 6-MF exhibits negligible activity against non-mycobacterial species (35). This discrepancy can be explained by the poor aqueous solubility of 6-MF. In our broth microdilution assay, the antimicrobial activity of 6-MF could not exceed its maximum soluble concentration, whereas in disk diffusion, local high concentrations at the agar surface may have led to an overestimation of its actual antibacterial activity.

Although 6-MF complies with Lipinski’s rule of five and its MIC against *M. abscessus* is below the maximum soluble concentration, its poor aqueous solubility may pose a significant obstacle to its development as an antibiotic. The addition of glycoside moieties may become one of the breakthroughs to overcome this solubility limitation, and we plan to explore this approach in our future work (36).

The need for narrow-spectrum antibiotics has long been emphasized in NTM therapy, owing to the risks of cross-resistance and microbiome disruption by broad-spectrum agents (37). The selectivity of 6-MF toward mycobacteria represents a desirable therapeutic strategy that minimizes off-target effects and may reduce resistance emergence among unrelated bacterial species.

In conclusion, 6-MF is a selective antimycobacterial agent that exhibits bacteriostatic activity and is associated with disruption of membrane energetics, characterized by membrane potential dissipation. 6-MF not only exhibited significant *in vitro* activity against *M. abscessus* but also showed *in vivo* efficacy in mice. In addition, 6-MF exhibited low resistance frequency and synergistic potential with clarithromycin, supporting its further development as a therapeutic candidate for *M. abscessus* infection.

## MATERIALS AND METHODS

### Chemistry

Unless noted otherwise, all starting materials and reagents were obtained from commercial suppliers (Strem chemicals, Aldrich, Acros Organics, Alfa Aesar, and TCI) and used without further purification. All solvents used for routine isolation of products and chromatography were reagent grade. Round-bottom flasks for reaction were dried at 80°C before use. Analytical thin‐layer chromatography (TLC) was performed using Merck silica gel glass plates with an F‐254 indicator and visualized by UV light (254 nm and 365 nm). Flash column chromatography was performed using silica gel 60 (230–400 mesh) with the indicated solvents. NMR spectra were obtained using a Varian VNMRS500 spectrometer (500 MHz for ^1^H NMR and 126 MHz for ^13^C NMR). ^1^H and ^13^C chemical shifts are reported in parts per million (ppm, *δ*) relative to TMS (tetramethylsilane), with the residual solvent peak used as an internal reference. Signals are reported as *m* (multiplet), *s* (singlet), *d* (doublet), *t* (triplet), *q* (quartet), dd (doublet of doublets), and ddd (doublet of doublet of doublet); coupling constants (*J*) are reported in hertz (Hz). High‐resolution mass spectrometry (HRMS) data were obtained with a Jeol AccuTOF (JMS‐T100TD) equipped with a DART (direct analysis in real‐time) ion source from Ionsens, (Tokyo, Japan) in ESI mode and JEOL AccuTOF (JMS‐T2000GC) instrument in EI mode.

### Synthesis of 6‐methyl‐2‐phenyl‐4H‐chromen‐4‐one (6-methylflavone)

To a 10 mL round-bottom flask, 6‐methyl‐4‐chromanone (50 mg, 0.308 mmol), Pd(TFA)_2_ (15 mg, 0.046 mmol), 5‐nitro‐1,10‐phenanthroline (21 mg, 0.092 mmol), phenyl boron pinacol ester (189 mg, 0.924 mmol), and anhydrous DMSO (1 mL) were added, and the mixture was heated at 100°C under an O_2_ (balloon) atmosphere for 48 h. The reaction was monitored by thin layer chromatography (TLC). After completion, the mixture was cooled to room temperature and extracted with EtOAc. The organic phase was dried with Na_2_SO_4_ and concentrated under reduced pressure. The residue was purified with flash column chromatography to afford 6‐methyl‐2‐phenyl‐4*H*‐chromen‐4‐one (6-MF) as a pale yellow solid (yield: 85%, 62 mg); ^1^H NMR (500 MHz, CDCl_3_) *δ* 8.03 (m, 1H), 7.96–7.91 (m, 2H), 7.56–7.51 (m, 4H), 7.48 (d, *J* = 8.5 Hz, 1H), 6.83 (s, 1H), 2.48 (s, 3H); HR‐MS: (ESI+) m/z [M+H]^+^ calcd for C_16_H_12_O_2_, 237.0910; found, 237.0919.

### Bacterial strains and growth conditions

All mycobacteria were grown in Middlebrook 7H9 broth supplemented with 10% albumin-dextrose-catalase (ADC), 2.5% glycerol, and 0.02% Tween-80, after removal of bacterial clumps for liquid cultures. For solid cultures, mycobacteria were streaked or plated on Middlebrook 7H10 agar plates supplemented with 10% oleic acid-albumin-dextrose-catalase (OADC) and 0.5% glycerol. Liquid cultures were incubated in a shaking incubator, and solid cultures were incubated in a stationary incubator. *M. marinum* and *M. paragordonae* were incubated at 30°C, and the others were incubated at 37°C.

Bacteria and yeast other than mycobacteria, including *A. baumannii*, *B. subtilis*, *C. albicans*, *E. coli*, *M. luteus*, *P. aeruginosa*, and *S. aureus*, were grown in Luria-Bertani (LB) broth at 37°C.

The bacterial and fungal strains used in this study are as follows: *M. avium* subsp. *avium* ATCC 25291, *M. bovis* BCG Tokyo, *M. chelonae* subsp. *bovistauri* QIA-37, *M. chelonae* subsp. *gwanakae* MOTT36W, *M. fortuitum* subsp. *fortuitum* ATCC 6841, *M. intracellulare* subsp. *yongonense* 05-1390^T^, *M. marinum* ATCC 927, *M. paragordonae* 49061^T^, *M. paraintracellulare* MOTT64, *A. baumannii* ATCC 17978, *B. subtilis* ATCC 6051, *C. albicans* NCCP 32557, *E. coli* NCCP 14541, *M. luteus* ATCC 4698, *P. aeruginosa* NCCP 14570, and *S. aureus* NCCP 14780.

All clinical isolates of *M. abscessus* were isolated from patients diagnosed with refractory pulmonary disease caused by *M. abscessus* infection and were provided by the Seoul National University Hospital.

### Flavonoid library synthetic strategy

The flavonoid library used in this study was chemically synthesized through organic synthesis. The privileged flavones were synthesized through the oxidative palladium(II)‐catalyzed dehydrogenation and sequential boron Heck reaction from chromanone (38). Using a similar method, Pd(II)‐catalyzed *β*‐arylation of chromanone with aryl boronic acid afforded various flavanones (39). Finally, aza-flavanones were obtained through Pd(II)‐catalyzed oxidative aza‐Michael cyclization from N‐Tf 2′‐aminodihydrochalcone (40). These synthetic approaches indicated that a novel flavonoid library was constructed efficiently with mild reaction conditions from commercially available starting materials.

### Construction of bioluminescent strains and plasmid

pMV306_LuxG13 plasmid was a gift from Brian Robertson and Siouxsie Wiles (Addgene plasmid #26161; http://n2t.net/addgene:26161; RRID: Addgene_26161) and was utilized in this study.

All bioluminescent mycobacterial strains were transformed with the indicated plasmid through the electroporation method as follows. Electrocompetent mycobacteria were prepared by washing with 10% glycerol thrice and subsequently electroporated with 3 µg of the plasmid using Gene Pulser II electroporation apparatus (Bio-Rad) under the following condition: 2.5 kV, 10 μF, 1,000 Ω, and a 0.1 cm gap. Transformants were selected on Middlebrook 7H10 agar plates supplemented with 10% OADC, 0.05% glycerol, and 100 μg/mL kanamycin. The selected colonies were then transferred to 7H9 broth medium supplemented with 10% ADC, 0.2% glycerol, 0.05% Tween-80, and 100 μg/mL kanamycin and cultured for 3–4 days. Once the transformants were cultured successfully, they were transferred to a 96-well white flat cell culture plate (SPL, Pocheon, Korea), and luminescence was measured using a Tecan Infinite F200 microplate reader.

### Cell culture

The murine macrophage cell line J774A.1 was purchased from Korean Cell Line Bank (Seoul, Korea). J774A.1 cells were cultured in RPMI-1640 medium supplemented with 10% fetal bovine serum (FBS) and 100 U/mL penicillin/streptomycin and were maintained at 37°C in a humidified atmosphere with 5% CO_2_ to ensure optimal growth and viability.

### Direct exposure procedure

Bioluminescent mycobacterial strains were grown to the stationary phase, washed with PBS, centrifuged, and resuspended in 7H9 broth supplemented with 10% ADC, 2.5% glycerol, and 0.02% Tween-80 to OD_600_ = 0.2. The prepared mycobacteria were loaded in a 96-well white flat cell culture plate. Subsequently, an equivalent volume of 7H9 broth containing the drug was added into the same plate. The luminescence was measured at 24 h using a Tecan Infinite F200 microplate reader.

### J774A.1 infection procedure

All mycobacterial strains were cultured until the stationary phase, washed with PBS, centrifuged, and then resuspended in RPMI-1640 supplemented with 10% FBS after removal of bacterial clump. J774A.1 cells were then infected with the specified mycobacteria at the multiplicity of infection (MOI) of 10 for 2 h at 37°C in a humidified atmosphere with 5% CO_2_. Following the infection, the cells were washed with PBS to remove extracellular mycobacteria. Subsequently, the cells were incubated in fresh culture medium containing 50 μg/mL amikacin for 1 h to eliminate any remaining extracellular mycobacteria. After the removal of amikacin-containing medium, the infected J774A.1 cells were treated with drug in RPMI-1640 supplemented with 2% FBS. The luminescence was measured at 24 h using a Tecan Infinite F200 microplate reader.

For the CFU assay, the infected cells were lysed with 1% Triton X-100 for 10 min. Subsequently, the lysates were serially diluted in PBS and plated on 7H10 agar plate supplemented with 10% OADC and 0.5% glycerol. The agar plates were cultured at 37°C until the colonies appeared.

### Neutral red uptake assay

The cytotoxicity of the drugs was evaluated through the neutral red uptake (NRU) assay. J774A.1 cells were treated with drug in transparent 96-well cell culture plate and incubated 48 h at 37°C in a humidified atmosphere with 5% CO_2_. The cells were then washed with PBS and stained with complete RPMI-1640 containing 100 μg/mL neutral red (Sigma) for 2 h at 37°C. The stained J774A.1 cells were destained with the destain buffer (50% ethanol, 49% distilled water, and 1% glacial acetic acid), and the absorbance was measured at 540 nm with Tecan Infinite F200 microplate reader to evaluate cell viability.

### Determination of MIC and MBC

MIC was determined using a broth microdilution assay according to CLSI guideline (41). Bacterial culture was washed with PBS and resuspended in cation-adjusted Mueller-Hinton broth (CAMHB) to a McFarland standard of 0.5. The resuspended cultures were further diluted in CAMHB with the ratio 1:100 and loaded into 96-well culture plates. Drugs were serially diluted in a 2-fold manner and loaded into the same 96-well plate. The plates were incubated at 37°C, and the absorbance was measured at 600 nm using a Tecan F200 microplate reader. The absorbance values measured in blank wells (media + drug at the same concentration) were subtracted from the absorbance value of test wells (media + drug + bacteria), and the MICs were determined as the lowest concentration that inhibited more than 90% of bacterial growth compared to non-treated control well.

MIC was also determined using a resazurin-based microtiter assay. Resazurin stock solution (150 μg/mL) was added to each well at 10% of the assay volume following broth microdilution assay, and plates were incubated at 37°C for 2 h protected from light. The fluorescence was measured at ex540/em590 nm using a Tecan F200 microplate reader. The RFU value measured in blank wells (media + drug + resazurin) was subtracted from the RFU value of test wells (media + drug + bacteria + resazurin), and the MICs were determined as the lowest concentration that inhibited more than 90% of bacterial growth compared to non-treated control wells.

For MBC determination, the bacterial cultures from the 96-well plate used for a broth microdilution assay were plated on Mueller-Hinton agar plate and incubated at 37°C for 3 days. The MBC was determined as the lowest concentration at which no colonies were observed on the plate. If the MBC/MIC ratio exceeds 4, the drug was considered a bacteriostatic agent; if the MBC/MIC ratio is lower than 4, the drug was considered a bactericidal agent.

### Checkerboard assay and the evaluation of synergy

For FICI evaluation, *M. abscessus* was grown until the stationary phase, washed with PBS, centrifuged, and resuspended in CAMHB to a McFarland standard of 0.5. The resuspended culture was further diluted in CAMHB with the ratio 1:100 and loaded into 96-well plates. Drugs were serially diluted in a 2-fold in CAMHB and loaded into the same 96-well plate in a left-to-right gradient, with low concentrations on the left and high concentrations on the right. Likewise, the other drug was loaded into the same 96-well plate in a gradient arrangement from top to bottom, with low concentrations at the top and high concentrations at the bottom. The plates were incubated at 37°C for 1 week, and the absorbance was measured at 600 nm using a Tecan Infinite F200 microplate reader. MIC was determined as the minimum concentration that inhibited ≥ 90% growth, and the FICI was calculated using the formula: $\frac{MIC_{A+B}}{MIC_{A}}+\frac{MIC_{A+B}}{MIC_{B}}$, where *MIC*_*A+B*_ represents the MIC values of drug A and drug B in combination. The interpretation of FICI values was as follows: FICI ≤ 0.5, synergy; 0.5 < FICI ≤ 4, indifference; and FICI > 4, antagonism (42).

For the J774A.1 infection model, the cells were infected with bioluminescent *M. abscessus* at an MOI of 10, as previously described, and incubated at 37°C in humidified air containing 5% CO_2_. Luminescence was measured 24 h post-infection using a Tecan F200 microplate reader. The growth inhibition rate of each well was calculated using the formula: $\left(1-\frac{test\;well}{non-treated\;control\;well}\right)\times 100\left(\%\right)$. The results were uploaded to SynergyFinder (https://synergyfinder.fimm.fi/), a web application interpreting drug interaction. The data were interpreted using four synergy scoring models: ZIP, Bliss, Loewe, and HSA. The synergy score for a drug combination was averaged over all the dose combination measurements, and the interpretation of synergy score was as follows: δ-score ≥ 5, synergy; −5 < δ-score < 5, indifference; δ-score ≤ 5, antagonism (43).

### Adaptive laboratory evolution assay

A broth microdilution assay was performed in 96-well culture plates using *M. abscessus* type strain, ATCC 19977, according to CLSI guideline, as previously described. The plates were incubated at 37°C for 7 days, and the MIC was determined based on the absorbance measured at 600 nm. The culture from the well containing the highest drug concentration in which bacterial growth was still observed was harvested and transferred to the next cycle of a broth microdilution assay. This process was repeated for five consecutive cycles.

### Membrane potential measurement using DiOC_2_(3)

*M. abscessus* ATCC 19977 was grown to the mid-log phase, washed with PBS, centrifuged, and resuspended in PBS containing 0.05% Tween-80 and 30 μM diOC_2_(3) to an OD_600_ of 0.5. The suspension was incubated at 37°C with shaking for 40 min. Subsequently, 80 μL of the bacterial suspension was loaded into each well of black 96-well plate, and the fluorescence was measured at ex485/em520 nm (green) and ex485/em620 nm (red) every 2 min for four consecutive measurements. Plates were then removed from the reader, and 20 μL of each compound was added to each well, followed by fluorescence measurement under the same conditions. The RFU value measured in blank wells (media + drug + diOC_2_(3)) was subtracted from the RFU value of the test well (media + drug + bacteria + diOC_2_(3)). Membrane potential was expressed as the red (620 nm) to green (520 nm) fluorescence ratio.

### Resazurin assay

*M. abscessus* ATCC 19977 was grown to the stationary phase, washed with PBS, centrifuged, and resuspended in CAMHB to an OD_600_ of 0.5. The bacterial suspension was seeded into a 96-well plate, and drugs were treated to each well. Resazurin stock solution was then added to a final working concentration (0.15 mg/mL) immediately, and fluorescence was monitored at ex540/em590 nm using a Tecan F200 microplate reader.

### Oxygen consumption rate measurement in *M. abscessus*

One day before the assay, a Seahorse XFe24 sensor cartridge was rehydrated overnight in a utility plate filled with XF calibrant at 37°C in a non-CO_2_ incubator. Separately, an XF24 cell culture plate was coated with 50 μg/mL poly-D-lysine at 4°C overnight.

Cultures of *M. abscessus* ATCC 19977 were washed with PBS and resuspended in unbuffered 7H9 medium. The bacterial suspension (10^6^ CFU/well) was seeded into the poly-D-lysine–coated XF24 cell culture plate and centrifuged at 2,000 × *g*. After confirming homogeneous bacterial adherence under a light microscope, the plate was further incubated for 30 min at 37°C in a non-CO_2_ incubator. The bacteria-loaded XF24 plate was then assembled with the rehydrated sensor cartridge and analyzed using a Seahorse XFe24 Analyzer.

### ATP measurement

ATP levels were determined using BacTiter-Glo microbial cell viability assay kit (Progema). *M. abscessus* ATCC 19977 was seeded to an OD_600_ of 0.2 in CAMHB containing each drug and incubated at 37°C with shaking. Aliquots of bacterial culture were collected at various time points and stored at −80°C until analysis. Each sample was loaded into a LumiNunc 96-well white plate (Thermo Fisher Scientific) and reacted with an equal volume of Bactiter-Glo reagent in the dark for 20 min. ATP standards ranging from 10 to 100 nM were also included in each assay to calculate ATP concentrations. Luminescence was measured using a Tecan F200 microplate reader.

### EtBr efflux assay

*M. abscessus* ATCC 19977 was grown to the mid-log phase, washed with PBS, centrifuged, and resuspended in PBS containing 0.05% Tween-80 and 2 μg/mL of EtBr to an OD_600_ of 0.5. The suspension was incubated at 25°C with shaking for an hour. Cells were then washed with ice-cold PBS and resuspended in the same volume of ice-cold PBS supplemented with 0.4% glycerol and glucose. Then, 100 μL of the suspension was loaded into a black 96-well plate, and an equal volume of each compound was added to the wells. Plates were immediately transferred to a plate reader prewarmed to 37°C, and fluorescence was measured at ex530/em590 nm every 2 min.

### Intracellular pH comparison using BCECF-AM

*M. abscessus* ATCC 19977 cultures were washed with PBS and resuspended in PBS containing 20 μM BCECF-AM to an OD_600_ of 0.8. The suspensions were incubated at 37°C with shaking in the dark and washed with PBS to remove extracellular dye. The bacteria were resuspended in PBS and loaded into a black 96-well plate. Compounds were then added to each well, and fluorescence was immediately measured at ex440/em535 nm or ex488/em535 nm using a Tecan F200 microplate reader.

The fluorescence intensity measured at ex488/em535 nm was divided by that measured at ex440/em 535 nm, and the resulting ratio was normalized to the DMSO control.

### PI uptake assay

*M. abscessus* ATCC 19977 cultures were washed with PBS and resuspended in unbuffered 7H9 broth to an OD_600_ of 0.5. The suspension was incubated at 37°C with shaking, and the 1 mL of the culture was harvested at 2, 6, and 24 h. The harvested culture was washed with PBS and resuspended in PBS containing 20 μM PI, and the suspension was incubated at 37°C for 15 min in the dark. Fluorescence was measured at ex535/em617 nm using a Tecan F200 microplate reader.

Considering that bacterial growth inhibition by 6-MF or inconsistent washing steps might affect the result, the PI-stained suspension was serially diluted in PBS and plated on 7H10 agar plates to determine CFU in the suspension (Table S2).

### ROS measurement using DCFDA

*M. abscessus* ATCC 19977 cultures were washed with PBS and resuspended in unbuffered 7H9 broth containing 40 μM DCFDA to an OD_600_ of 0.5. The suspensions were incubated at 37°C with shaking for 30 min and then washed with PBS three times to remove excess DCFDA. The bacteria were resuspended in fresh unbuffered 7H9 broth with or without bovine serum catalase (3 μg/mL) and transferred into 96-well plates containing 6-MF at serial 2-fold concentrations. The plate was incubated at 37°C for 30 min in the dark, and fluorescence was measured at ex485/em535 nm using a Tecan F200 microplate reader.

### Animal experiments

Female BALB/c mice (~20 g, 6 weeks old) were purchased from Orient-Bio (Seong-nam, South Korea). Mice were bred and housed in specific pathogen-free conditions at the Seoul National College of Medicine. The experiment was initiated following a 7-day acclimation period from the day mice arrived.

Cyclophosphamide at a dosage of 150 mg/kg was given to mice via intraperitoneal route on days 1 and 4 before infection. Mice were anesthetized with 2.5% isoflurane in a chamber and subsequently challenged with a clinical isolate of *M. abscessus* rough morphotype (10^6^ CFU) via the intranasal route on the following day. 6-MF and amikacin were then administered at a dose of 20 mg/kg via the intraperitoneal route daily from the following day of infection. Body weight of each mouse was monitored daily, beginning on the day of infection.

On the 14th day post-infection, mice were euthanized by cervical dislocation following anesthesia. A blood sample was collected from the retro-orbital sinus prior to euthanasia. Lungs were then excised from the mice. The postcaval lobe was fixed in 10% formalin at 4°C, while the other lobes were homogenized in PBS. The homogenates were subsequently serially diluted in PBS and plated on 7H10 agar plates supplemented with 10% OADC and 0.5% glycerol for counting CFU in lungs. These plates were incubated at 37°C until colonies appeared, and the colonies were then counted. The fixed postcaval lobes were embedded in a paraffin block and sectioned. These sectioned tissues were stained with hematoxylin and eosin and subsequently observed under a light microscope.

## Data Availability

All data generated or analyzed during this study are included in this article and supplemental materials.

## SUPPLEMENTAL MATERIAL

Supplemental material spectrum.03588-25-s0001.docx Tables S1 and S2; Fig. S1 to S3.

ASM does not own the copyrights to Supplemental Material that may be linked to, or accessed through, an article. The authors have granted ASM a non-exclusive, world-wide license to publish the Supplemental Material files. Please contact the corresponding author directly for reuse.
