# Supplementary material for: 6-methylflavone exerts selective antimycobacterial activity with membrane energetics disruption and demonstrates therapeutic potential against Mycobacterium abscessus infection
Source: Microbiol Spectr. 2026 Apr 20;14(6):e03588-25. doi: 10.1128/spectrum.03588-25 (PMC13228053; doi:10.1128/spectrum.03588-25)
Supplement: Supplemental material — Tables S1 and S2; Fig. S1 to S3. [file spectrum.03588-25-s0001.docx]

| **Clinical isolate** | **Subspecies** | **Morphotype** | **Resistance-related genes** | | | | **Susceptibility** | | |
| --- | --- | --- | --- | --- | --- | --- | --- | --- | --- |
|  |  |  | ***rrl*** | | ***erm(41)*** | ***rrs*** | **macrolides** | | **aminoglycosides** |
|  |  |  | **2058** | **2059** | **28** | **1408** | **intrinsic** | **inducible** |  |
| #1 | *abscessus* | Rough | A | A | T | A | susceptible | resistant | susceptible |
| #2 | *massiliense* | Rough | A | A | - | A | susceptible | susceptible | susceptible |
| #3 | *abscessus* | Smooth | A | A | T | A | susceptible | resistant | susceptible |

**Supplemental Table 1.** The list of clinical isolates used for gernerating bioluminescent reporter strains and their characteristics.

| **CFU/mL** | **DMSO** | **6-MF (8 μg/mL)** | **6-MF (16 μg/mL)** | **6-MF (32 μg/mL)** | **CCCP (25 μM)** |
| --- | --- | --- | --- | --- | --- |
| **2 h** | 3.0E+08 | 4.0E+08 | 2.5E+08 | 4.5E+08 | 3.5E+08 |
|  | 4.0E+08 | 5.0E+08 | 7.5E+08 | 1.5E+08 | 5.0E+08 |
|  | 3.0E+08 | 2.5E+08 | 6.5E+08 | 3.0E+08 | 5.5E+08 |
| **6 h** | 5.5E+08 | 1.4E+09 | 5.5E+08 | 7.0E+08 | 2.0E+08 |
|  | 5.5E+08 | 1.4E+09 | 5.0E+08 | 7.5E+08 | 1.0E+08 |
|  | 4.0E+08 | 1.7E+09 | 7.0E+08 | 6.5E+08 | 1.0E+08 |
| **24 h** | 4.0E+08 | 8.0E+08 | 5.0E+08 | 7.0E+08 | 1.0E+06 |
|  | 7.5E+08 | 5.0E+08 | 6.0E+08 | 5.0E+08 | 2.0E+06 |
|  | 1.2E+09 | 4.0E+08 | 4.5E+08 | 2.0E+08 | 4.5E+06 |

**Supplemental Table 2.** CFU measurements of bacetrial suspensions before fluorescence measurement in the PI uptake assay.


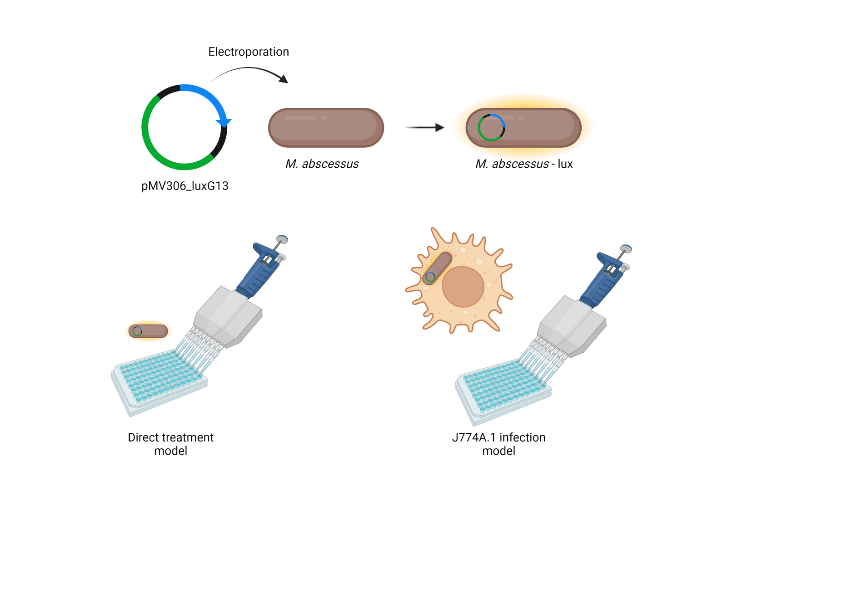


**A**

**C**

**B**

**C**

**Supplemental Figure 1.** The feasibility of bioluminescent *M. abscessus* for antimicrobial screening system. **(A)** Schematic diagram of production of Mab_luxG13. **(B)** Correlation between luminescence and OD_600_ in Mab_luxG13 growth culture. **(C)** Reduction of luminescence upon treatment of amikacin in Mab_luxG13 growth culture.

**Supplemental Figure 2.** MIC determination of 6-MF against diverse microorganisms other than mycobacteria. A broth microdilution assay was performed according to CLSI guideline, and the plates were incubated for 20 h. OD_600_ was measured using a Tecan F200 microplate reader (n=3). Error bars represents the SEM

**Supplemental Figure 3.** MICs of 6-MF and flavone against *M. abscessus* ATCC 19977. A broth microdilution assay was performed according to CLSI guideline, and the plates were incubated for 72 h. OD_600_ was measured using a Tecan F200 microplate reader (n=3). Error bars represents the SEM
